# Pressure ulcer multidisciplinary teams via telemedicine: a pragmatic cluster randomized stepped wedge trial in long term care

**DOI:** 10.1186/1472-6963-14-83

**Published:** 2014-02-24

**Authors:** Anita Stern, Nicholas Mitsakakis, Mike Paulden, Shabbir Alibhai, Josephine Wong, George Tomlinson, Ann-Sylvia Brooker, Murray Krahn, Merrick Zwarenstein

**Affiliations:** 1 ELLICSR, University Health Network, 200 Elizabeth Street, Munk Building, BCS021, Toronto, Ontario M5G 2C4, Canada; 2 THETA, Leslie Dan Faculty of Pharmacy, University of Toronto, 144 College StreetToronto, Ontario M5S 3 M2, Canada; 3 Faculty of Medicine & Dentistry, University of Alberta, 736 University Terrace, 8303 112 St, Edmonton, AB T6G 2 T4, Canada; 4 Toronto General Hospital, Eaton 14th Floor Rm 14EN214, 200 Elizabeth St., Toronto, Ontario M5G 2C4, Canada; 5 Toronto General Hospital, Eaton North Wing, 13th Floor Rm 13EN238, 200 Elizabeth St., Toronto, Ontario M5G 2C4, Canada; 6 Centre for Studies in Family Medicine, Department of Family Medicine, Schulich School of Medicine & Dentistry, Western University, 245-100 Collip Circle, Research Park, London, Ontario N6G 4X8, Canada

**Keywords:** Pressure ulcer, Long term care, Nursing home, Multi-disciplinary team, Chronic wound, Treatment

## Abstract

**Background:**

The study was conducted to determine the clinical and cost effectiveness of enhanced multi-disciplinary teams (EMDTs) vs. ‘usual care’ for the treatment of pressure ulcers in long term care (LTC) facilities in Ontario, Canada

**Methods:**

We conducted a multi-method study: a pragmatic cluster randomized stepped-wedge trial, ethnographic observation and in-depth interviews, and an economic evaluation. Long term care facilities (clusters) were randomly allocated to start dates of the intervention. An advance practice nurse (APN) with expertise in skin and wound care visited intervention facilities to educate staff on pressure ulcer prevention and treatment, supported by an off-site hospital based expert multi-disciplinary wound care team via email, telephone, or video link as needed. The primary outcome was rate of reduction in pressure ulcer surface area (cm^2^/day) measured on before and after standard photographs by an assessor blinded to facility allocation. Secondary outcomes were time to healing, probability of healing, pressure ulcer incidence, pressure ulcer prevalence, wound pain, hospitalization, emergency department visits, utility, and cost.

**Results:**

12 of 15 eligible LTC facilities were randomly selected to participate and randomized to start date of the intervention following the stepped wedge design. 137 residents with a total of 259 pressure ulcers (stage 2 or greater) were recruited over the 17 month study period. No statistically significant differences were found between control and intervention periods on any of the primary or secondary outcomes. The economic evaluation demonstrated a mean reduction in direct care costs of $650 per resident compared to ‘usual care’. The qualitative study suggested that onsite support by APN wound specialists was welcomed, and is responsible for reduced costs through discontinuation of expensive non evidence based treatments. Insufficient allocation of nursing home staff time to wound care may explain the lack of impact on healing.

**Conclusion:**

Enhanced multi-disciplinary wound care teams were cost effective, with most benefit through cost reduction initiated by APNs, but did not improve the treatment of pressure ulcers in nursing homes. Policy makers should consider the potential yield of strengthening evidence based primary care within LTC facilities, through outreach by APNs.

**Trial registration:**

ClinicalTrials.gov identifier NCT01232764

## Background

A pressure ulcer (PU), also known as a pressure sore, decubitus ulcer, or bedsore, is a localized injury to the skin and/or underlying tissue caused by pressure and/or shear [1]. Those at greatest risk for developing PUs are the elderly, the critically ill, the neurologically impaired, and those who suffer from conditions associated with immobility [2]. PUs are staged with a 4 point classification system denoting severity, ranging from stage I (intact skin, non-blanchable erythema), to stage IV (full thickness tissue loss exposing bone, tendon, or muscle) [1]. In 2007 the National Pressure Ulcer Advisory Panel added 2 categories of PUs: Deep Tissue Injury (DTI) (intact discolored skin due to damage to underlying soft tissue), and Unstageable (full-thickness tissue loss, depth unknown due to slough or eschar on the wound’s surface) [1].

Pressure ulcers (PUs) are problematic across healthcare settings throughout the world [3,4]. Financial costs associated with PUs are high [5,6]. Pain [7], depression [8], altered self-image [8], and increased morbidity and disability [9] are consequences of this largely preventable condition.

The Medical Advisory Secretariat (MAS) [10], a unit that prepares evidence-based analyses to support policy relevant decision-making by the Ontario Ministry of Health and Long-Term Care (MOHLTC), conducted a review of the literature leading them to suggest that an enhanced multi-disciplinary team (EMDT) to assess wound variables and determine optimal treatment would be effective for the treatment of PUs, but the composition of these teams, specific roles and responsibilities of members, and intensity of involvement were far less certain [11]. Service delivery by expert teams traditionally necessitates physical transport of patients to these teams, or vice versa. Telemedicine offers a means to deliver chronic wound care services remotely [12,13].

Due to the complexity of PUs, recommendations made by the MAS expert panel, high PU prevalence rates in Long Term Care (LTC) facilities in Canada [3], the paucity of high quality evidence related to pressure ulcer treatment [14], and the potential to enhance care delivery using telemedicine we conducted the following study to evaluate the clinical and cost effectiveness of an EMDT supported by telemedicine vs. 'usual' care (UC) for the treatment of PUs in LTC.

The study was funded by the MOHLTC, the Canadian Patient Safety Institute, and the Central Community Care Access Centre. ClinicalTrials.gov identifier: NCT01232764.

## Methods

### Quantitative

#### Design

We conducted a multi-method study: a pragmatic stepped-wedge cluster- randomized trial, ethnographic observation and in-depth interviews, and an economic evaluation. A stepped wedge design [15] was selected for the trial in order to retain the power of randomization while offering all facilities enrolled in the trial exposure to what was believed to be a desirable intervention and to enable delivery of the intervention to these facilities by a small study team.

#### Population

The study population was residents with Stage II or greater PUs residing in eligible LTC facilities situated within 2 geographic regions (Local Health Integration Networks-LHINs) in southern Ontario, Canada.

#### Eligibility was assessed at 3 levels: LTC facility, resident, and wound

Facilities were eligible and approached for the study by the principal investigator if they had a minimum of 100 beds, were within 100 kilometers from the hospital housing the expert wound team, had a PU prevalence greater than the provincial average (5.5%, based on data collected from LTC facilities in Ontario by the Canadian Institute of Health Research in 2009), and the facility administrator provided consent. The criteria of a minimum of 100 beds was applied in order to maximize the reach of the study staff, while ensuring that average sized facilities in Ontario (170 beds) were represented in the study. The criteria of facilities being within 100 km of the hospital housing the wound care team was applied to allow for the physical transport of residents to the wound care team if deemed necessary as determined by the study referral rubric. The criteria of greater than provincial average PU prevalence rates was applied since these are the facilities within which the intervention would be situated if the intervention was found to be effective.Residents in consenting LTC facilities were eligible if they had a reported PU (stage II or greater), and provided informed consent. Their legal representative was approached for consent if the resident was deemed incapable by the most responsible clinician. Facility staff obtained verbal consent to release names of eligible residents to the research assistants. Research assistants were responsible for participant recruitment. Individuals were approached to participate after facilities had been randomised.

If a patient had more than one wound, all were included. Stage I and Deep Tissue Injury PUs were excluded as skin typically remains intact, and are therefore not amenable to accurate measurement of wound surface area.

Facility enrollment in the 17- month study occurred in October 2010. Residents were recruited from participating facilities from October 2010-February 2012, with data collection closing in March 2012.

The study was approved by the research ethics boards of the University of Toronto and the hospital housing the acute care wound team.

#### Randomisation

Randomisation was at the level of the facility (cluster) due to the educational component of the intervention being delivered at the facility level. LTC facilities were randomised to start date of the intervention by a researcher external to the study team using a computer-generated random number sequence, giving each LTC facility different lengths of control and intervention periods (Table 1).

**Table 1:** Study design

**Time (Months)**		**1**	**2**	**3**	**4**	**5**	**6**	**7**	**8**	**9**	**10**	**11**	**12**	**13**	**14**	**15**	**16**	**17**
**Facility number**	**12**	-	-	-	-	-	-	-	c	c	c	c	c	c	**P1**	**P1**	**P1**	**P2**
	**11**	-	-	-	-	-	-	c	c	c	c	c	**P1**	**P1**	**P1**	**P2**	**P2**	**P2**
	**10**	c	c	c	c	c	c	c	c	c	c	c	c	**P1**	**P1**	**P1**	**P2**	**P2**
	**9**	c	c	c	c	c	c	c	c	c	c	c	**P1**	**P1**	**P1**	**P2**	**P2**	**P2**
	**8**	c	c	c	c	c	c	c	c	c	c	**P1**	**P1**	**P1**	**P2**	**P2**	**P2**	**P2**
	**7**	c	c	c	c	c	c	c	c	c	**P1**	**P1**	**P1**	**P2**	**P2**	**P2**	**P2**	**P2**
	**6**	c	c	c	c	c	c	c	c	**P1**	**P1**	**P1**	**P2**	**P2**	**P2**	**P2**	**P2**	**P2**
	**5**	c	c	c	c	c	c	c	**P1**	**P1**	**P1**	**P2**	**P2**	**P2**	**P2**	**P2**	**P2**	**P2**
	**4**	c	c	c	c	c	c	**P1**	**P1**	**P1**	**P2**	**P2**	**P2**	**P2**	**P2**	**P2**	**P2**	**P2**
	**3**	c	c	c	c	c	**P1**	**P1**	**P1**	**P2**	**P2**	**P2**	**P2**	**P2**	**P2**	**P2**	**P2**	**P2**
	**2**	c	c	c	c	**P1**	**P1**	**P1**	**P2**	**P2**	**P2**	**P2**	**P2**	**P2**	**P2**	**P2**	**P2**	**P2**
	**1**	c	c	c	**P1**	**P1**	**P1**	**P2**	**P2**	**P2**	**P2**	**P2**	**P2**	**P2**	**P2**	**P2**	**P2**	**P2**

c = Control; **P1** = Intervention- Phase 1 (onsite one day/week); **P2** = Intervention Phase 2 (primarily remote bi-weekly).

#### Intervention: Enhanced Multi-Disciplinary Team (EMDT)

Every LTC facility in both groups appointed a wound care lead (RN or RPN) at study start to serve as the primary contact for the study team. The control period ranged from 3-12 months per facility as determined by randomisation order (Table 1). Blinding of facility staff or residents was not possible due to the nature of the intervention.

**Figure 1:**
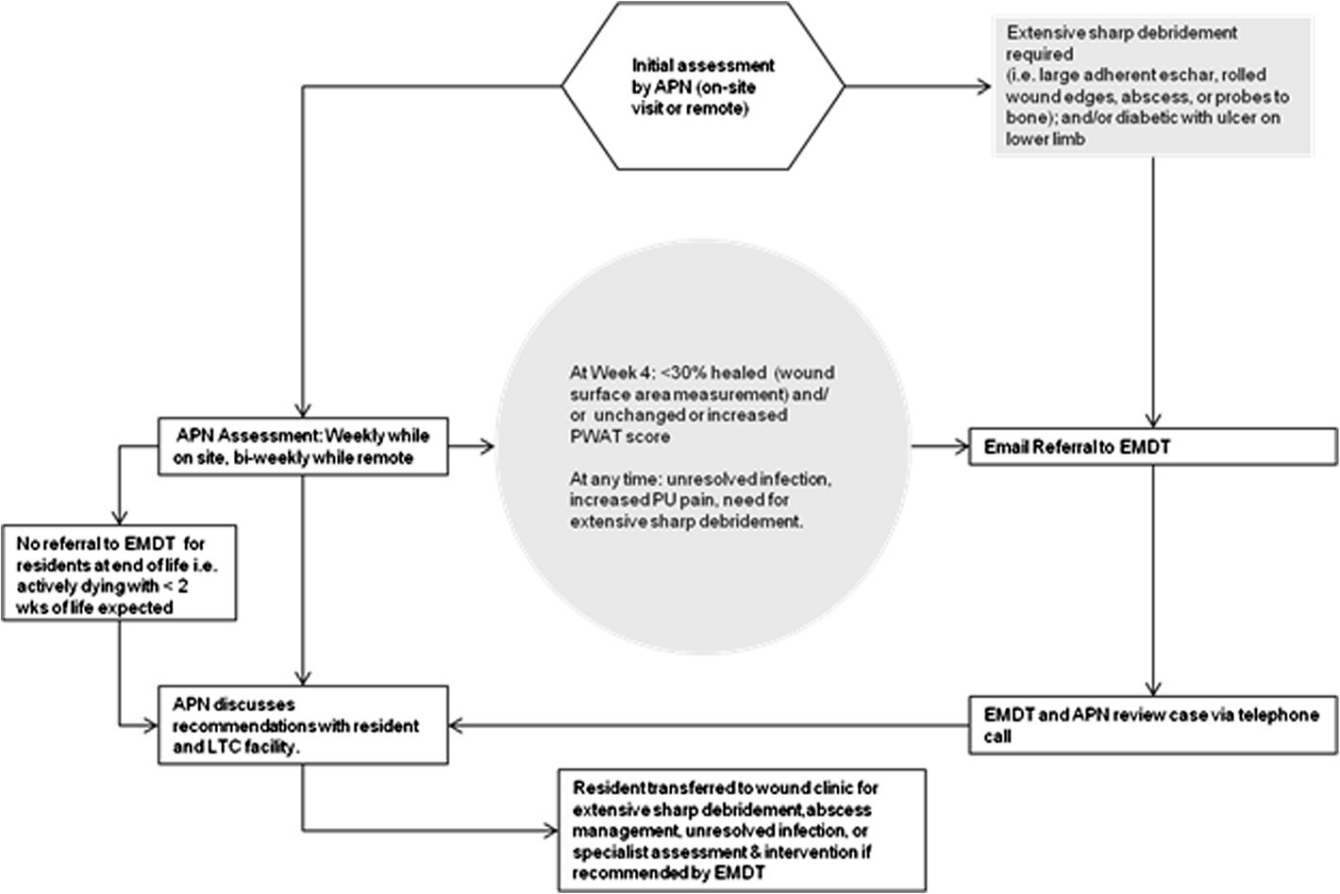
Referral rubric.

The Enhanced Multi-Disciplinary Team (EMDT) consisted of advanced practice nurses (APNs) who provided outreach to LTC facilities, and were linked to a hospital based expert wound care team. The APNs, funded by the study budget, had expertise in skin and wound care (two were certified enterostomal therapists with over 5 years of experience, one was a masters prepared nurse with over 10 years of experience in wound care). They visited LTC facilities to educate staff on the prevention and treatment of pressure ulcers, consulting with a hospital based expert wound care team via email, telephone, or video link following a referral rubric (Figure 1). The expert wound team was situated in a large teaching hospital. It was led by a nurse practitioner and included a chiropodist, an occupational therapist, and a plastic surgeon that had ready access to a wide variety of additional hospital based specialists if consultations were deemed necessary.

The intervention was implemented in 2 phases at each LTC facility. Phase 1 was 3 months in length at each facility and focused on training of LTC staff. APNs spent one day per week at each facility to establish multi-disciplinary wound care teams within facilities, and to educate direct care staff (personal support workers, nursing assistants, registered nurses) about PU prevention and treatment (case based teaching at the bedside, and structured group sessions customized to meet facility’s needs). All recommendations were based on the Registered Nurses’ Association of Ontario (RNAO) evidence based guidelines (updated in 2007) [16] contextualized to the LTC setting. In addition APNs worked directly with a facility wound care lead, mentoring them in the assessment of wounds enrolled in the study, digital wound photography, and completion of standardized assessment and treatment forms, liaising with the expert team following a referral rubric developed for the study to reflect real world practice (Figure 1).

Phase 2 (1-11 months in length) involved primarily remote support of the facility wound care lead by the APNs via email and telephone. The wound care lead was to assess PUs, complete assessment and treatment forms, take digital photos, and transmit de-identified data via email to the APNs every 2 weeks. APNs reviewed cases with the wound care lead via telephone and email, reviewing referral criteria with them, and consulting with the expert team accordingly. APNs would visit the facility when necessary or if requested to do so by the facility wound care lead. This process was repeated every 2 weeks for all PUs until healed, or until the end of the study period, whichever came first. Face to face (or video-link) visits with the expert team occurred after review of individual cases as indicated by the referral rubric.

#### Control: "Usual" care

Wound care within LTC facilities was typically provided by registered nurses (RNs), registered practical nurses (RPNs), personal support workers (PSWs), and nutritionists, who may or may not have had expertise in wound care. Although facilities were to have wound care teams in place within the facility as mandated by the MOHLTC in 2010, only 3 of the 12 facilities had wound care teams, with the composition and function of these ‘teams’ being highly variable. Access to other disciplines (e.g. Enterostomal Therapists, Physiotherapists, Occupational Therapists) was available, typically on a reactive basis. Wound care specialists who were requested by facilities to assess and treat advanced wounds, costs that were covered by the MOHLTC upon facility application for reimbursement, were typically employed by wound product companies, or were in private practice and reimbursed on a fee-for-service basis.

#### Outcome measures

The primary outcome was rate of reduction in PU surface area (cm^2^/day), judged by an external study staff member blinded to facility allocation who was trained in wound surface area measurement captured by digital photography. Research assistants visited each facility every two weeks to obtain digital photos of PUs. Measurement of all photos was delayed until 2 months prior to study completion to decrease risk of un-blinding the assessor to facility allocation as the stepped wedge design enabled identification of random allocation at the beginning and end of the study period. Wounds were measured from digital photos using Adobe Photoshop CS5 [17]. The Quick Selection Tool and Histogram were used to estimate the size of each wound in pixels. The length (in pixels) of the paper ruler visible in each photo was then measured, allowing each estimated wound size to be converted to cm^2^.

Secondary outcomes were: time to complete healing (days), percentage of wounds healed, PU incidence, PU prevalence, wound pain (Visual Analogue Scale-Pain), hospitalization, emergency department visits, utility(EQ5D), and cost effectiveness. Research assistants administered surveys every 2 weeks throughout the study period (EQ5D, VAS-Pain). The EQ5D and VAS-Pain were administered to mentally competent residents. The EQ5D was also measured by proxy (the clinician most familiar with each enrolled resident). PUs were followed until healed, or until the end of the study period, whichever came first. A research assistant, trained in chart abstraction, abstracted data from resident’s facility charts at the end of the study period to capture hospital admissions, emergency department visits, and cost.

#### Sample size estimation

We simulated data under a stepped wedge design over a 17‒month study duration using normalized wound sizes in a hierarchical linear model with between‒ulcer variation in healing rates (a mean of –0.0865 (per week) and a standard deviation of 0.038). The mean was chosen to reflect the 12‒week mean healing time of Stage II pressure ulcers [18], while the standard deviation reflects a wide range of ulcer healing trajectories that included the possibility of observing ulcers that are increasing in size up to a maximum of 200% over the study duration. We used a between‒facility standard deviation of 0.02 [19] in the distribution of the treatment effects, in a stepped wedge design with between five and 10 facilities, 170 patients per facility, and a 20% drop‒out rate applied at baseline, as well as measurement error of normalized wound surface areas (0.1 standard deviation units). The percentage of ulcers that were estimated to not respond to the intervention was 20%, the prevalence of Stage II‒IV ulcers was 4%, and the annual incidence of Stage II‒IV pressure ulcers was 2.5% [20]. After discussion with wound care clinical experts that included a plastic surgeon, a nurse practitioner, enterostomal therapists, a chiropodist, and advance practice nurses, we estimated the minimal clinically important difference to be a 40% improvement in the normal rate of healing (8.65% per week), which corresponded to an absolute healing rate of 12.11% per week. Using the R statistical software (version 2.9.1), we simulated 1000 datasets under the stepped wedge design for each combination of the number of facilities and the treatment effect. The power was estimated as the proportion of significant treatment effects, across the 1000 simulated datasets. 80% power was considered adequate. Because the effect of the treatment is estimated by comparing the pre and post-intervention healing rates within each facility, one issue important to the design of standard cluster randomized trials does not apply here: the relative sizes of the between-facility and within-facility variation in healing rates (i.e., the ICC) were unimportant. The between-facility variation in healing rates is captured by the facility-specific random effect for rate and only the absolute size of the within-facility variance is important. Sets of simulations with different values for the between-facility variance all gave essentially the same simulated power. Based on the above assumptions a study with 10 facilities had 80% power to detect a 40% increase in the healing rate, under a 5% significance level.

#### Statistical methods

Demographic and clinical characteristics of residents participating in the study, as well as the characteristics of the PUs observed during the study, were described with means and standard deviations for continuous variables and frequencies and percentages for categorical variables.

Healing rate was analyzed with the use of linear mixed effects models [21], using log-transformed PU area as outcome and intervention, time and their interaction, along with a number of covariates (wound stage, Charlson Comorbidity Index, PU recurrence, bed bound, and urinary or fecal incontinence) as predictors. The covariates were selected based on previous evidence of effect on PU healing [18]. Mixed effects models were used to account for the hierarchical clustering structure of the data (multiple measurements per PU, PUs per patient, patients per facility). PU stage specific subgroup analyses were also performed. After testing for the proportional hazard assumption, time to healing was analyzed with Cox Proportional Hazard frailty models [22], using the same covariates as those for the analysis of the primary outcome. Six months probability of healing was estimated using Kaplan-Meier unadjusted method [23]. Incidence rates were estimated and compared between control and intervention periods using a random effects model accounting for the heterogeneity between facilities [24]. Prevalence was estimated and compared between the two periods with the use of logistic mixed effects models. Rates of hospitalization and emergency department visits were analyzed with the use of negative binomial regression models. Linear mixed effects models were also used for the analysis of wound related pain and EQ5D utilities (measuring quality of life) outcomes, after adjusting for age and other confounders (sex, diabetes, BMI). All analyses were performed using the R statistical software (version 2.14.0).

### Qualitative evaluation

Purposive sampling (maximum variation) was employed to select facilities in which to conduct ethnographic observation and in-depth interviews, with variation in the following characteristics that may influence PU healing rates: facility ownership (i.e. for profit vs. non-profit), staff turnover, PU prevalence rates, and length of exposure to the intervention. Facilities were also selected to ensure variation in facility size and location (i.e. LHIN). Interviews were audiotaped and transcribed verbatim by a professional transcriptionist. APNs kept field notes related to staff perceptions of, and experiences with the intervention throughout the intervention period at every facility (n = 12). Qualitative data were not collected during the control period to minimize the presence of study personnel, and minimize the potential for a Hawthorne effect.

Data collection and analysis followed an iterative process. A coding scheme was developed and subsequently revised in order to account for new themes and concepts that arose from the re-reading of the transcripts. Key concepts and an analytic framework were developed that interpreted and accounted for the empirical data. Data were obtained from multiple sources to ensure rigour and data quality: researcher observation, in-depth and informal interviews, APN observations, and bedside audit data. Ethnographic observation was conducted on multiple floors in facilities and at various times throughout the day and evening to increase understanding of organizational context. The iterative collection and analysis of data ensured that we could test hypotheses, or explore unexpected findings as they emerged in the analysis. In addition, the report was sent to the APNs for feedback and their comments incorporated.

### Economic evaluation

A comprehensive paper describing the methods used for the economic evaluation can be found elsewhere [25]. The primary analysis estimated the change in direct care costs associated with introducing 'enhanced' multi-disciplinary wound care teams (EMDTs) compared to 'usual' care (UC) for the treatment of pressure ulcers in LTC facilities in Ontario. The perspective of each analysis was that of the Ontario Ministry of Health and Long-Term Care (MOHLTC). The time horizon was the time until residents were first in a wound-free state or were censored. Costs were reported in 2012 Canadian dollars.

## Results

### Quantitative

#### Facility and resident recruitment

**Figure 2:**
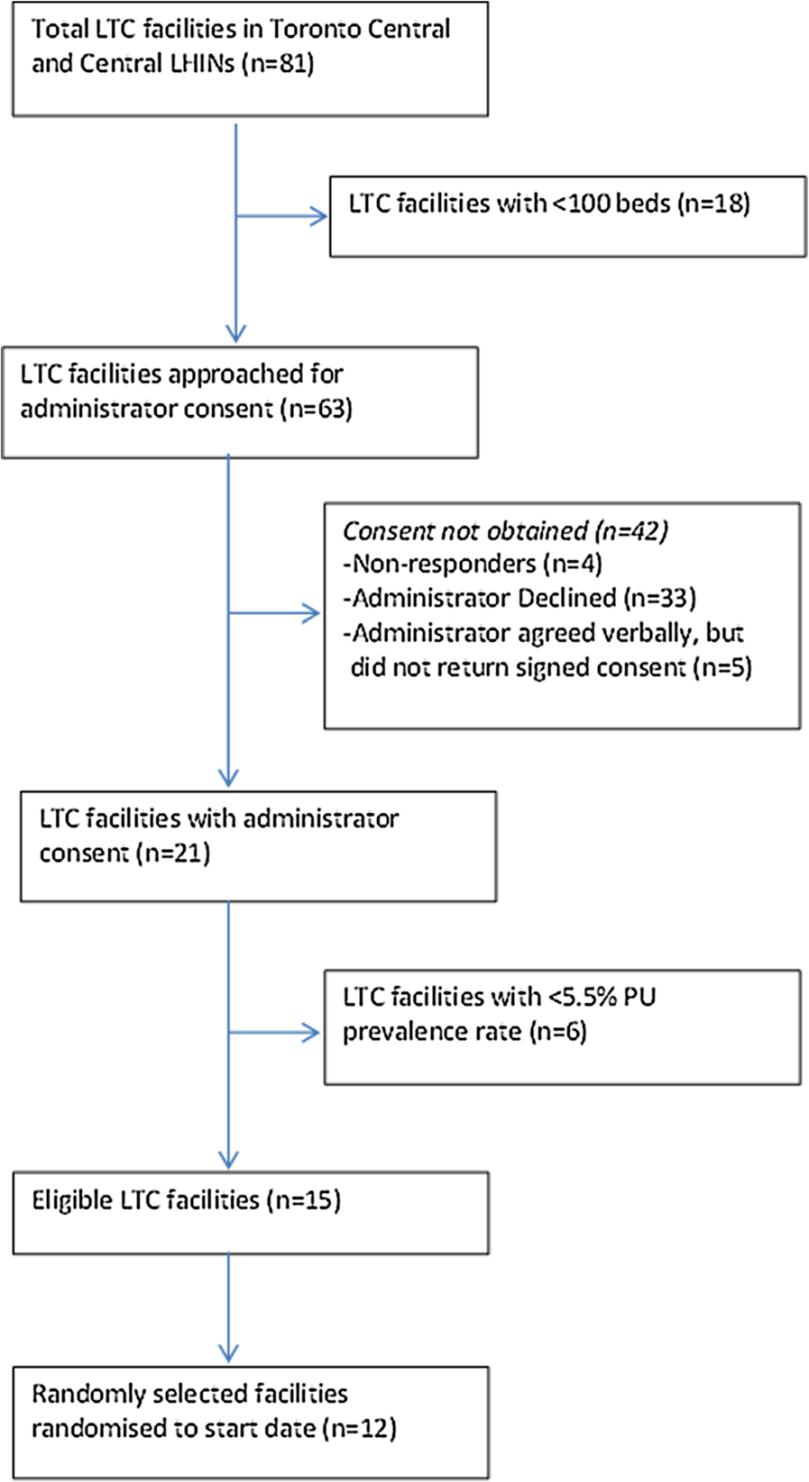
Facility recruitment.

Ten facilities were randomly selected to participate in the study from among 15 consenting eligible facilities. Prevalence rates were lower than anticipated (Table 2), and so 2 additional eligible facilities were randomly selected from the eligible sites and randomized to start date of the intervention in consultation with a statistician to ensure integrity of the study design. Therefore 12 eligible facilities in total, each with an average of 166 beds (SD = 37.1), were randomized (Figure 2).

**Table 2:** PU prevalence

**LTC facility**	**PU prevalence (%) based on Q42009 MDS from CIHI**	**PU prevalence (%) based on PUs reported to study staff at study start (i.e. Oct 2010 for facilities 1-10; April 2011 for facility 11; May 2012 for facility 12)**	**PU prevalence (%) based on PUs reported to study staff 2 wks. prior to Intervention start**	**PU prevalence (%) based on PU identification by APNs at Intervention start (i.e. bedside audit)**	**PU prevalence (%) based on PUs reported to study staff at study end**
**1**	9.9	4.4	6.2	6.8	4.4
**2**	10.2	5.9	2.5	3.5	2.0
**3**	5.9	3.3	9.2	9.2	8.3
**4**	7.0	4.4	1.0	3.9	0**
**5**	8.4	5.6	2.5	2.5	0
**6**	6.3	3.8	2.9	3.8	0.8
**7**	7.9	2.4	2.4	5.7	3.3
**8**	6.8	0.6	0.6	5.7	4.4
**9**	12.3	2.5	6.0	6.0	4.3
**10**	6.7	1.9	3.8	3.8	4.4
**11**	9.5	6.3	3.1	4.4	3.1
**12**	6.8	3.7	2.1	2.1*	1.6
**Mean(SD)**	**8.1 (1.9)**	**3.7 (1.7)**	**3.5 (2.5)**	**4.7 (2.0)**	**3.0 (2.4)**

*7residents excluded from bedside audit as requested by Director of Care.

**Director of Care stopped referring residents with PUs to the study APN during phase 2 of the Intervention.

**Figure 3:**
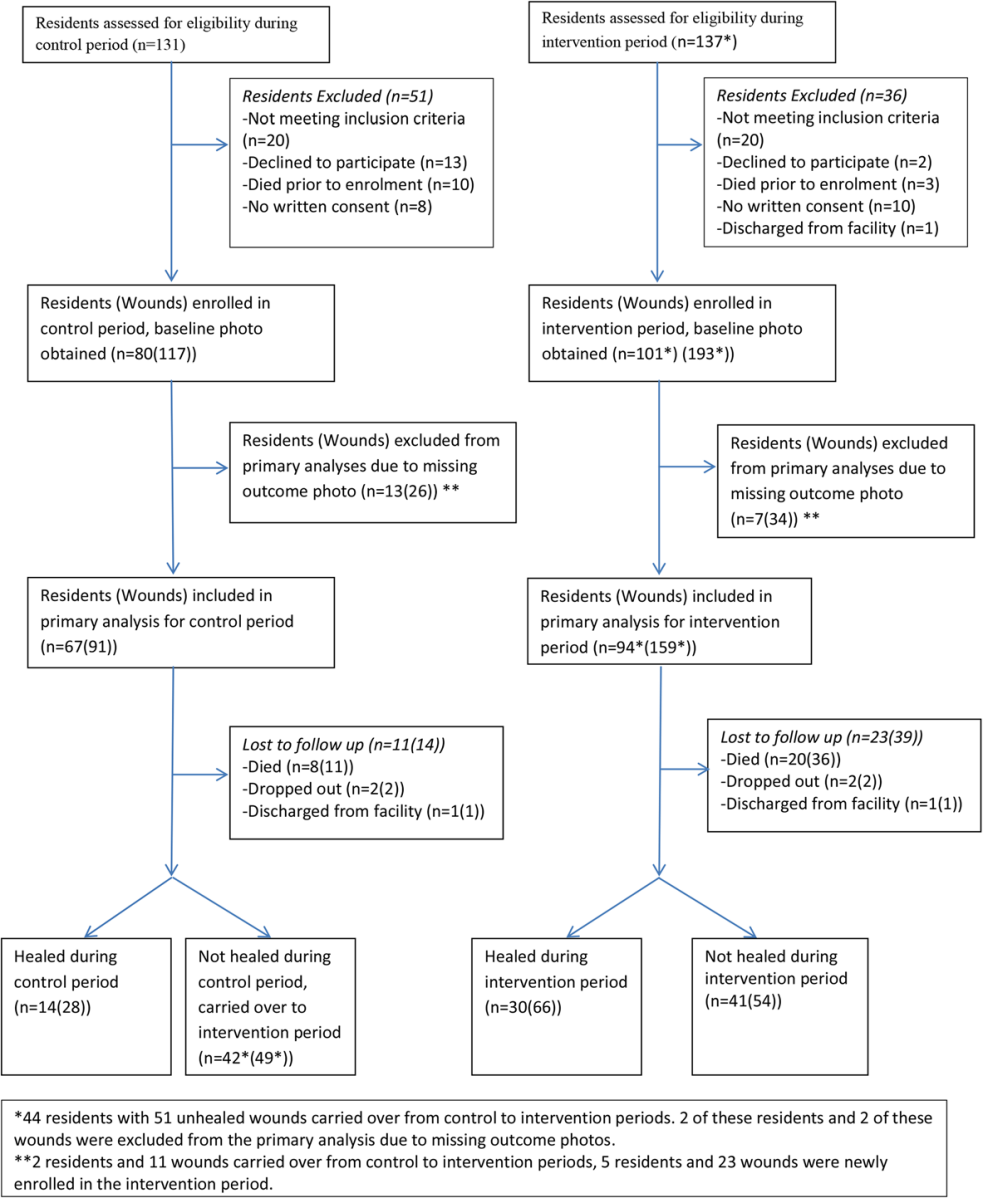
Resident (wound) recruitment.

137 residents with a total of 259 PUs were recruited from these 12 facilities during the 17-month study period (Figure 3) (Table 3).

**Table 3:** Participant characteristics

**Variable**	Control (N = 67*)	Intervention (N = 94*)
**Age**	81 (12)	83 (12)
**Sex (Female)**	43 (64.2%)	65 (69.1%)
**Charlson co-morbidities**		
Alzheimer's/Dementia	38 (56.7%)	62 (66.0%)
Diabetes	22 (32.8%)	36 (38.3%)
Stroke/TIA	20 (29.9%)	29 (30.9%)
Diabetes with end organ damage	10 (14.9%)	22 (23.4%)
Paraplegia/Hemiplegia	11 (16.4%)	15 (16.0%)
Any solid tumour	6 (9.0%)	16 (17.0%)
COPD	10 (14.9%)	7 (7.4%)
Congestive Heart Failure	4 (6.0%)	11 (11.7%)
Peripheral Vascular Disease	5 (7.5%)	6 (6.4%)
Myocardial Infarction	3 (4.5%)	6 (6.4%)
Moderate or severe renal disease	1 (1.5%)	4 (4.3%)
Charlson Co-morbidity Index	3 (2)	3 (2)
**Other co-morbidities**		
Hypertension	37 (55.2%)	55 (58.5%)
Osteoarthritis	36 (53.7%)	42 (44.7%)
Osteoporosis	23 (34.3%)	33 (35.1%%)
Coronary Artery Disease	15 (22.4%)	18 (19.1%)
Parkinson's disease	7 (10.4%%)	9 (9.6%)
Contractures	5 (7.5%)	6 (6.4%%)
Spasticity	4 (6.0%)	5 (5.3%)
**PU Risk factors**		
Incontinence (urine)	66 (98.5%)	91 (96.8%)
Incontinence (stool)	58 (86.6%)	79 (84.0%)
Bedbound	49 (81.7%%)	71 (87.7%)
Mental Status (Not alert/not oriented)	52 (77.6%)	78 (83.0%)
Nutritional supplement	55 (82.1%)	80 (85.1%)
Tube Feed	5 (7.5%)	7 (7.4%)
Body Mass Index	25 (8)	24 (6)

*42 participants crossed study phases, extending from control to intervention i.e. double counted.

#### Outcomes

##### Primary outcome

Fifty-six of the 259 PUs were excluded from analysis due to having only one measurement. Wounds in two additional photos were deemed ‘unmeasurable’. Therefore, measurements of 201 PUs from 119 residents were used in the analyses of the primary outcome.There was no difference in the rate of healing with and without the intervention, with the average rate of healing being 1.0058 times slower in the intervention period (95%CI = 0.985-1.027,p = 0.60) (Table 4).

**Table 4:** Primary analysis – healing rate

**Description of model**	**Pre-intervention slope (β) NB: negative values indicate healing**	**Effect of intervention**			
		**Change in slope (Δ**_**0**_**) NB: values < 0 indicate benefit**	**Relative effect on healing NB: values < 1 indicate benefit**		**p-value**
			**Estimate**	**95% CI**	
Random effect for intercept and slope, common treatment effect, wound stage, CCI, recurrence, bedbound, any incontinence	-0.116	0.0055	1.006	0.985-1.027	0.605
Random effect for intercept and slope, common treatment effect*	-0.114	0.0062	1.006	0.985-1.027	0.539
Random effect for intercept slope, and treatment effect*	-0.122	0.0171	1.020	0.993-1.042	0.161
Random effect for intercept and slope, common treatment effect, control for stage at diagnosis	-0.115	0.0053	1.006	0.985-1.026	0.615
Models run by stage*					
Stage II: Random effect for intercept and slope, common treatment effect	-0.116	-0.040	0.968	0.882-1.062	0.079
Stage III: Random effect for intercept and slope, common treatment effect	-0.126	0.005	1.005	0.958-1.055	0.828
Stage IV: Random effect for intercept and slope, common treatment effect	-0.125	0.050	1.050	1.014-1.088	0.006
Unstageable: Random effect for intercept and slope, common treatment effect	-0.158	0.013	1.013	0.972-1.056	0.534

*indicates unadjusted analysis.

When analyzed by stage, a similar pattern was found, with the exception of stage IV PUs where there was a clinically unimportant, but statistically significant decrease in healing rates (rate of healing was 1.05 times slower post-intervention, 95% CI = 1.014-1.088, p = 0.0063).

An analysis that compared ulcers treated entirely before the intervention, with those treated entirely after the intervention, and with those ulcers that were identified before the intervention but which were also treated in the intervention period showed no differences in outcome (Table 5).

**Table 5:** Sensitivity analysis of healing rates

**Group**	**Healing rate (Standard error)**	**Difference in healing rates (Standard error) NB: negative values indicate benefit**	**Relative effect on healing (95% CI)**	**p-value**
**(a) Pre-intervention**	-0.156 (0.087)	0.050 (0.094)	1.051 (0.874-1.263)	0.5916
**(b) Post-intervention**	-0.106 (0.030)			
**(c) Pre-and post-intervention**	N/A	-0.070 (0.047)	0.932 (0.850-1.022)	0.1363

##### Secondary outcomes

All secondary outcomes showed a similar pattern, with no benefit and wide confidence intervals.

#### Time to healing

From the 91 PUs identified during the control period, 28(30.8%) healed in the control period, and 3 (3.32%) were censored at the end of the control period. There were 49(53.86%) wounds whose care continued to the intervention period of which 24 healed (49.0%) while 25 were censored (51.0%) at the end of the study. From the 110 PUs newly enrolled during the intervention period 42 (43%) healed during the intervention period while 81 (57%) did not heal and were censored. Table 6 reports the results of a proportional hazards model of wound healing, using pre-specified covariates as described in the Statistical Methods section. Our survival model shows the same result as the primary analysis, as does our pre-planned subgroup analyses by wound stage (Table 7): there is no statistically significant benefit associated with the intervention. Facility-level effects were also explored and differences between facilities were found (Table 8).

**Table 6:** Hazard ratios, time to healing

**Variable**	**Hazard ratio (95% CI)**	**p-value**
**Intervention**	1.48 (0.79,2.78)	0.22
**Log(initial PU area)**	0.65 (0.51,0.83)	0.00045
**Para/hemiplegia or CVA**	0.76 (0.43,1.36)	0.36
**CCI = 3-5 vs. CCI = 0-2**	1.54 (0.90,2.64)	0.12
**CCI ≥ 6 vs. CCI = 0-2**	0.68 (0.31,1.49)	0.34
**Recurrent PU**	1.55 (0.41,5.85)	0.52

CCI = Charlson Co-morbidity Index.

CVA = Cerebrovascular accident.

**Table 7:** Time to healing by wound stage

**Wound stage**	**Hazard ratio (95% CI)**	**p-value**
**II**	1.02 (0.40,2.63)	0.96
**III**	2.16 (0.87,5.34)	0.096
**IV**	1.58 (0.20,12.67)	0.66
**Unstageable**	1.00 (0.24,4.17)	1.0

**Table 8:** Exponentiated random effects for the intervention covariate

**Facility**	**Intervention**
1	0.94
2	0.48
3	1.62
4	1.94
5	0.93
6	2.20
7	2.29
8	0.49
9	0.64
10	0.92
11	0.68
12	0.76

#### Percentage of Wounds Healed

The percentage of wounds healed was evaluated as the probability of healing at 6 months (n = 201) using non parametric Kaplan-Meier estimators [22], allocating healing events (numerator) and exposure time (denominator) to the appropriate study period. The estimation was done separately for the control and intervention periods, without adjustment for PU stage. The probability of healing for the control period was estimated to be 35.0% (22.4, 45.6), and for the intervention period to be 53.4% (41.4, 62.9). This difference is not statistically significant as the two confidence intervals overlap.

#### Incidence rate

The model estimated the incidence rate of the intervention to be 1.12 (0.74, 1.68) times larger than the incident rate of the control (p = 0.59). Therefore, the model did not provide evidence of a significant difference of the incidence rate between the control and the intervention periods. Additionally, the model did not identify a significant heterogeneity among the facilities, I-sq = 10%, p = 0.38.

#### Wound pain

Mean VAS wound-specific pain scores were estimated to be 0.39 units higher during the intervention period, but the difference was not significant (p = 0.42, 95% CI = -055, 1.34).

#### PU prevalence

The logistic mixed effects model estimated the mean prevalence of residents with PUs to be equal to 2.22% (1.79%, 2.76%) in the control period vs. 2.40% (1.81%, 3.19) in the intervention period. The difference is not statistically significant (p = 0.6).

#### Hospitalizations

The mean hospitalization rate was estimated to be 1.2 (0.62, 2.36) times larger during the intervention period than during the control period. The difference was not statistically significant (p = 0.59).

#### Emergency department (ED) visits

The mean ED visit rate was estimated to be 1.3 (0.58, 2.90) times larger during the intervention period than during the control period. The difference was not statistically significant (p = 0.52).

#### Utility

Mean utilities were estimated to be 0.03 (-0.029, 0.088) units lower during the intervention period than during the control period. The difference was not statistically significant (p = 0.32).

#### Process measure: expert wound team referrals

Thirty seven of the 137 residents (27%) met criteria for referral to the expert team. Twenty five of the 37 residents (68%) were actually referred to the team, with a total of 28 consults. Twelve of the 37 residents (32%) were not referred to the EMDT despite meeting referral criteria; two of the 12 not referred to the EMDT were seen by specialists situated in hospitals adjacent to the LTC facilities, one APN felt facility lack of adherence to treatment recommendations made referrals for 4 residents futile, while no reason was cited for non-referral of 6 residents.The Nurse Practitioner attended all consultations, the chiropodist attended 16 (57%), the OT attended 13 (46%), the plastic surgeon attended 3 (11%), and an orthopedic surgeon attended 1. A recommendation for change in treatment resulted from 7 of the 28 consults (25%). Most consults occurred by email followed by a telephone call (n = 25, 89%). 2 consults were face to face at the hospital based wound clinic, and 1 consult occurred remotely via video-link.

Data abstracted from resident’s charts revealed one case of referral to a hospital based plastic surgeon and chiropodist during the control period. This LTC facility was located directly beside a large teaching hospital.

### Qualitative

The qualitative study results are presented in full elsewhere [25]. In summary, data collection and analyses from in-depth interviews and observation conducted in 5 of the 12 facilities, in addition to interviews conducted with members of the expert wound team in the hospital, and with the study APNs, revealed the critical role facility leadership played in ensuring implementation of the intervention. Although the intervention was well received in most facilities, inadequate allocation of staff time to its implementation, and unavailability of the required wound care supplies, prohibited effective uptake of the intervention across most facilities.

### Economic

The economic study results are presented in full elsewhere [25]. In summary, EMDTs were estimated to reduce direct care costs by $649 per resident, (Table 9) although this estimate was subject to substantial uncertainty. This was driven by cancellations of prescribed Negative Pressure Wound Therapy (NPWT) by the outreach APNs, and thus reduced costs, offset by an increase in costs related to increased hospital admissions in the intervention period.

**Table 9:** Comparison of direct care costs incurred until healing

**Cost category**	**UC**	**EMDTs**	**Difference**
*Personnel costs*			
Study nurse	N/A	$101	$101
MDT	N/A	$20	$20
ET	$357	$18	-$340
Facility nurse	$1,094	$1,486	$392
*Total personnel costs*	*$1,451*	*$1,624*	*$173*
*Treatments and supplies costs*			
Antibiotics	$84	$38	-$46
Dressings	$1,623	$2,284	$661
NPWT	$3,142	$0	-$3,142
*Total treatments and supplies costs*	*$4,849*	*$2,322*	*-$2,527*
*Hospital costs*			
Inpatient	$4,147	$5,792	$1,645
Ambulatory (ER)	$250	$310	$60
*Total hospital costs*	*$4,397*	*$6,102*	*$1,705*
**Grand Total**	**$10,697**	**$10,048**	**-$649**
*Grand total (w/o Dressings costs)*	*$9,074*	*$7,764*	*-$1,310*
*Grand total (w/o NPWT costs)*	*$7,555*	*$10,048*	*$2,493*
*Grand total (w/o Hospital costs)*	*$6,300*	*$3,946*	*-$2,354*
*Grand total (w/o Dressings, NPWT costs)*	*$5,932*	*$7,764*	*$1,832*
*Grand total (w/o Dressings, Hospital costs)*	*$4,677*	*$1,662*	*-$3,015*
*Grand total (w/o NPWT, Hospital costs)*	*$3,158*	*$3,946*	*$788*
*Grand total (w/o Dressings, NPWT, Hospital costs)*	*$1,535*	*$1,662*	*$127*

## Discussion

The study was powered to detect a 40% difference in the primary outcome, rate of healing. Our findings show no significant improvement in rate of healing, with a narrow confidence interval. None of the secondary outcomes showed significant benefit. In spite of its clinical equivalence, the economic evaluation provided some evidence of a cost reduction. This was largely attributed to the discontinuation of NPWT on all residents in the intervention period. This decision by outreach APNs was appropriate as there is no evidence of effectiveness of this therapy for the treatment of pressure ulcers [26]. This resulted in a net savings of $649/resident. Thus although not superior in clinical outcomes, EMDTs may nonetheless be cost effective. Arguably, though, there would be much simpler ways to discourage use of this single, extremely expensive and unproven treatment.

Although the hospital based expert wound team played an important role for a small number of participants, and virtual communication between facilities, APNs, and the expert wound team was feasible, this study illustrates the relatively minor role played by this team for the treatment of PUs for residents in this study.

Our qualitative work suggests that, even in these potentially atypical consenting facilities, the intervention was not well embedded due to frequent staff turnover and insufficient managerial attention, suggesting that widespread implementation of this intervention under present conditions of LTC management would be challenging. Given these difficulties, and the high levels of satisfaction and engagement by LTC staff with the outreach APN when present in the LTC facility, we suggest that the intervention be more likely to succeed given a longer period of implementation, and that the whole of this be conducted using face to face APN support, rather than remote, approaches. This would of course, substantially raise the cost, perhaps eliminating the current benefit.

Although we adjusted for known predictors of wound healing, and there is no evidence explicitly linking advanced age and poor health status to wound healing, residual differences in these factors may have impaired wound healing in the intervention period, so our estimates of effectiveness may be somewhat pessimistic.

This study was limited to one particular expert wound care team located within a large teaching hospital and may not be representative of all expert multi-disciplinary wound care teams. 21 of 63 (33.3%) facility administrators agreed to participate. Therefore the facilities that participated in this study may not be a representative sample, but both of these potential biases would be expected to increase the effectiveness of the intervention.

We observed a large proportion of censored observations (n = 107, 53.2%), which reduces the effective sample size of the analysis, potentially introducing bias for the estimate of the time to healing, resulting in potentially inaccurate approximations of the variances used in the analysis. Additionally, any occurrence of uneven censoring due to uneven lengths of observation time between the two periods may potentially introduce bias in the estimation of the difference of the healing time between the two periods. However, the narrow confidence interval around clinically insignificant results suggests that this issue may not be important.

We adhered to the recently published methodological recommendations for comparative effectiveness research on chronic wounds [27], including the conduct of an economic evaluation to inform health care policy decision making. Studies of various wound treatments and products abound, typically sponsored by product companies, however studies addressing health service delivery models for wound care are limited.

Our study results are aligned with other studies that show no impact of specialist teams- in this case pharmacist/wound care nurse- on healing PUs on residents in LTC [28], and others that highlight the importance of effective leadership in LTC [29,30], consequences of high rates of staff turnover in these settings [31-33], and challenges in meeting residents’ needs within the current resource constraints. The feasibility of conducting wound consults remotely is also supported by the literature [34,35].

## Conclusions

PUs tend to be largely overlooked as LTC facility staff are busy reacting to more pressing issues (eg. disruptive behaviors, falls), and due to incentives in the reimbursement policies in place at the time this study was conducted, facilities could get extra costs related to wound care covered by the MOHLTC (e.g. costs for NPWT) once residents had advanced PU’s, reducing the incentive to address wounds proactively.

Incentives to prevent wounds across health care sectors might be needed as a precursor to interventions such as EMDTs. Our study findings suggest that policy makers should shift their focus away from specialty multi-disciplinary wound care teams external to facilities and direct their attention towards strengthening evidence based primary care within LTC. Use of outreach APNs to increase the capacity of existing staff in LTC to prevent wounds may be a more sustainable and effective model, providing effective management teams are in place and APNs are readily available to facilities for an extended period of time.

Future research is needed to increase our understanding of multi-disciplinary team functioning in LTC especially in relation to improved in-house capacity to prevent wounds. Based on this new knowledge, interventions could then be developed and evaluated to improve the organization and delivery of primary care in these settings which would in turn support appropriate and timely referral to external specialty wound care teams.

## Abbreviations

APN: Advanced practice nurse; CIHI: Canadian Institutes of Health Research; EMDT: Enhanced multi-disciplinary team; LHIN: Local health integration network; LTC: Long term care; MDT: Multi-disciplinary team; MDS: Minimum data set; MOHLTC: Ministry of health and long term care; PSW: Personal support worker; PWAT: Photographic wound assessment tool; PU: Pressure ulcer; RN: Registered nurse; RPN: Registered practical nurse; UC: Usual care; VAS: Visual analogue scale.

## Competing Interests

The authors declare that they have no competing interests.

## Authors contributions

AS, MZ, SA, MK, and NM contributed to study conception and design. JW and ASB contributed to data collection. GT and NM conducted all statistical analyses. All authors contributed to data analysis and interpretation of data. All authors reviewed and approved the manuscript.

## Pre-publication history

The pre-publication history for this paper can be accessed here:

http://www.biomedcentral.com/1472-6963/14/83/prepub
